# Vascular CXCR4 Expression – a Novel Antiangiogenic Target in Gastric Cancer?

**DOI:** 10.1371/journal.pone.0010087

**Published:** 2010-04-08

**Authors:** Barbara Ingold, Eva Simon, Ute Ungethüm, Ralf-Jürgen Kuban, Berit M. Müller, Amelie Lupp, Ulf Neumann, Matthias P. A. Ebert, Carsten Denkert, Wilko Weichert, Stefan Schulz, Christoph Röcken

**Affiliations:** 1 Institute of Pathology Campus Mitte, Charité University Hospital, Berlin, Germany; 2 Laboratory of Functional Genomics, Charité University Hospital, Berlin, Germany; 3 Institute of Pharmacology and Toxicology, Friedrich-Schiller-University, Jena, Germany; 4 Department of General, Visceral and Transplantation Surgery, Campus Virchow, Charité University Hospital, Berlin, Germany; 5 Department of Medicine II, Klinikum rechts der Isar, Technical University, Munich, Germany; The University of Hong Kong, Hong Kong

## Abstract

**Background:**

G-protein-coupled receptors (GPCRs) are prime candidates for novel cancer prevention and treatment strategies. We searched for differentially expressed GPCRs in node positive gastric carcinomas.

**Methodology/Principal Findings:**

Differential expression of GPCRs in three node positive vs. three node negative intestinal type gastric carcinomas was analyzed by gene array technology. The candidate genes CXCL12 and its receptor CXCR4 were validated by real-time reverse-transcription polymerase chain reaction in an independent set of 37 gastric carcinomas. Translation was studied by immunohistochemistry in 347 gastric carcinomas using tissue microarrays as well as in 61 matching lymph node metastases. Protein expression was correlated with clinicopathological patient characteristics and survival. 52 GPCRs and GPCR-related genes were up- or down-regulated in node positive gastric cancer, including CXCL12. Differential expression of CXCL12 was confirmed by RT-PCR and correlated with local tumour growth. CXCL12 immunopositivity was negatively associated with distant metastases and tumour grade. Only 17% of gastric carcinomas showed CXCR4 immunopositive tumour cells, which was associated with higher local tumour extent. 29% of gastric carcinomas showed CXCR4 positive tumour microvessels. Vascular CXCR4 expression was significantly associated with higher local tumour extent as well as higher UICC-stages. When expressing both, CXCL12 in tumour cells and CXCR4 in tumour microvessels, these tumours also were highly significantly associated with higher T- and UICC-stages. Three lymph node metastases revealed vascular CXCR4 expression while tumour cells completely lacked CXCR4 in all cases. The expression of CXCL12 and CXCR4 had no impact on patient survival.

**Conclusions/Significance:**

Our results substantiate the significance of GPCRs on the biology of gastric carcinomas and provide evidence that the CXCL12-CXCR4 pathway might be a novel promising antiangiogenic target for the treatment of gastric carcinomas.

## Introduction

Gastric cancer is one of the most common cancers worldwide, ranking fourth in overall frequency and accounting for over 650,000 deaths annually [1]. The mortality of gastric cancer is only excelled by lung cancer. Early gastric cancer often causes no specific symptoms. The lack of early symptoms delays the diagnosis. Consequently, 80–90% of Western patients with gastric cancer present with advanced tumours when local or distant metastases had already occured [1]. The lymph node status, especially the ratio of metastasis-positive/metastasis-negative lymph nodes, is the strongest prognostic factor of gastric cancer [2]. The 5-year survival rate for patients with 1–6 lymph node metastases is 44% and ending with only 11% in patients with more than 15 positive lymph nodes. Partial or total gastrectomy, combined with adjuvant radiotherapy and/or chemotherapy as indicated, promises complete cure only in patients with early stage disease. In metastatic disease, currently used radiotherapeutic and chemotherapeutic regimens have poor efficacy and treatment resistant disease progression leads to death within few months [3]. To date, there exists no specific predictive marker like HER2 in breast carcinoma, EGFR in non small cell lung carcinoma or K-RAS in colorectal carcinoma, which enables a more individualized therapeutic strategy. Therefore, new molecular-targeted therapeutic approaches are needed.

G-protein-coupled receptors (GPCRs) represent by far the largest family of cell-surface molecules, which relay signals via GTP-binding protein (G-protein) -initiated second messenger cascades into the cell [4]. GPCRs are regulated by many agonists, but all share a characteristic core composed of seven transmembrane α-helices, which are linked through three intra- and three extracellular loops. These receptors control key physiological functions, including neurotransmission, hormone and enzyme release from endocrine and exocrine glands, immune responses, muscle contraction and blood pressure regulation to name a few [4].

Malignant cells often hijack the normal physiological functions of GPCRs to survive, proliferate autonomously and evade the immune system. Furthermore GPCRs play a central role in tumour-induced angiogenesis and cancer metastasis. Many solid tumours rely on GPCRs to elicit an angiogenic response either by acting on endothelial or stromal components directly or through regulation of the release or activity of other angiogenic mediators such as vascular endothelial growth factor (VEGF) or basic fibroblast growth factor (bFGF) by stromal and immune cells [5]. Cancer cells manipulate GPCRs to attract endothelial cells and lead them to invade the tumour mass, thereby forming new vessels to provide nutrient and oxygen. Many cancers metastasize to specific organs, what frequently is caused by the aberrant expression of GPCRs in cancer cells - especially chemokine receptors - concomitant with the release of chemokines from the secondary organs [6].

Drug delivery, tumour imaging and biomarkers predicting malignancy are applications of GPCRs to highlight: Radio-labelled peptides that bind to GPCRs might have broad applications for cancer diagnosis and therapy [7]. Ligands that bind GPCRs have also been conjugated to cytotoxic agents to specifically target malignant cells that overexpress these receptors, therefore reducing side effects [8]. Furthermore, GPCRs might be valuable biomarkers for cancer diagnosis as proven by studies in prostate cancer [9].

Therefore, we aimed to (i) assess differentially expressed GPCRs in nodal negative versus nodal positive intestinal type gastric carcinoma by GeneChip array technique. (ii) Transcription of candidate genes was validated by real-time reverse-transcription polymerase chain reaction (real-time RT-PCR). We evaluated the translation and histoanatomical distribution of the chemokine CXCL12 and its corresponding chemokine receptor CXCR4 in a large series of 347 gastric carcinoma samples immunohistochemically using the tissue microarray-technology as well as in 61 matching lymph node metastases on conventional slides (iii). We correlated the translational expression patterns with an ample set of clinicopathological patient characteristics, including patient survival (iv).

## Results

### Differential gene expression in node negative and node positive gastric cancer tissue

First, we studied the differential expression of mRNA in a series of 6 intestinal type gastric cancer patients (3 with and 3 without lymph node metastases) using the GeneChip® Human Genome U133 Plus 2.0 Array from Affymetrix which detects 47,000 transcripts and variants as well as 38,500 well characterized human genes. mRNA was extracted and transcribed only from tissue samples obtained from the primary tumours. A total of 115 transcripts were found to be up- and 219 to be down-regulated in node positive gastric cancer compared with node negative gastric cancers (table S1). Next we searched for differentially expressed GPCRs. We identified 52 GPCRs and GPCR-related genes, which were up- or down-regulated with a fold change factor of >1.5 (table S2).

### *In silico* analysis

We then searched the *ENTREZ* data base of the “*National Center for Biotechnology Information (NCBI)*” for entries of the GPCRs and GPCR-ligands and their putative role in tumour biology. The significance of the angiotensin II type 1-receptor in gastric cancer was previously verified by our own group [10]. Several studies had shown that the expression of the duffy blood group chemokine receptor (DARC) correlated inversely with the prevalence and prognosis of prostate cancer [11]–[13]. In the animal model of breast cancer the expression of EDG2 correlated with the incidence of lung metastases [14]. FPRs mediate the effect of annexin 1 on cell motility and invasion, which are important for the metastatic potential of tumour cells [15]. LGR5 was recently shown to be a stem cell marker of cells of the small intestine and colon and stem-cell specific loss of Apc results in progressively growing neoplasias [16]. Collectively, these data provide evidence that our approach identified GPCRs and GPCR-ligands that may be involved in gastric cancer biology. Concerning chemokine receptors and chemokines, the expression of the chemokines CCL2 and CXCL12 were increased in nodal positive gastric cancer compared to nodal negative cases (supplementary table S2). Since the CXCL12-CXCR4 axis plays a prominent role in tumourigenesis, promoting angiogenesis and migration of tumour cells to metastatic sites [17]–[19], we selected CXCL12 and its receptor CXCR4 for further analyses.

### Transcription of CXCL12 and CXCR4 in gastric cancer

The differential expression of CXCL12- and CXCR4-mRNA was validated by real-time RT-PCR in an independent set of 37 intestinal type gastric carcinoma samples. We compared non-neoplastic mucosa with the primary tumour as well as primary tumours of node negative with primary tumours of node positive cancers.

**Figure 1:**
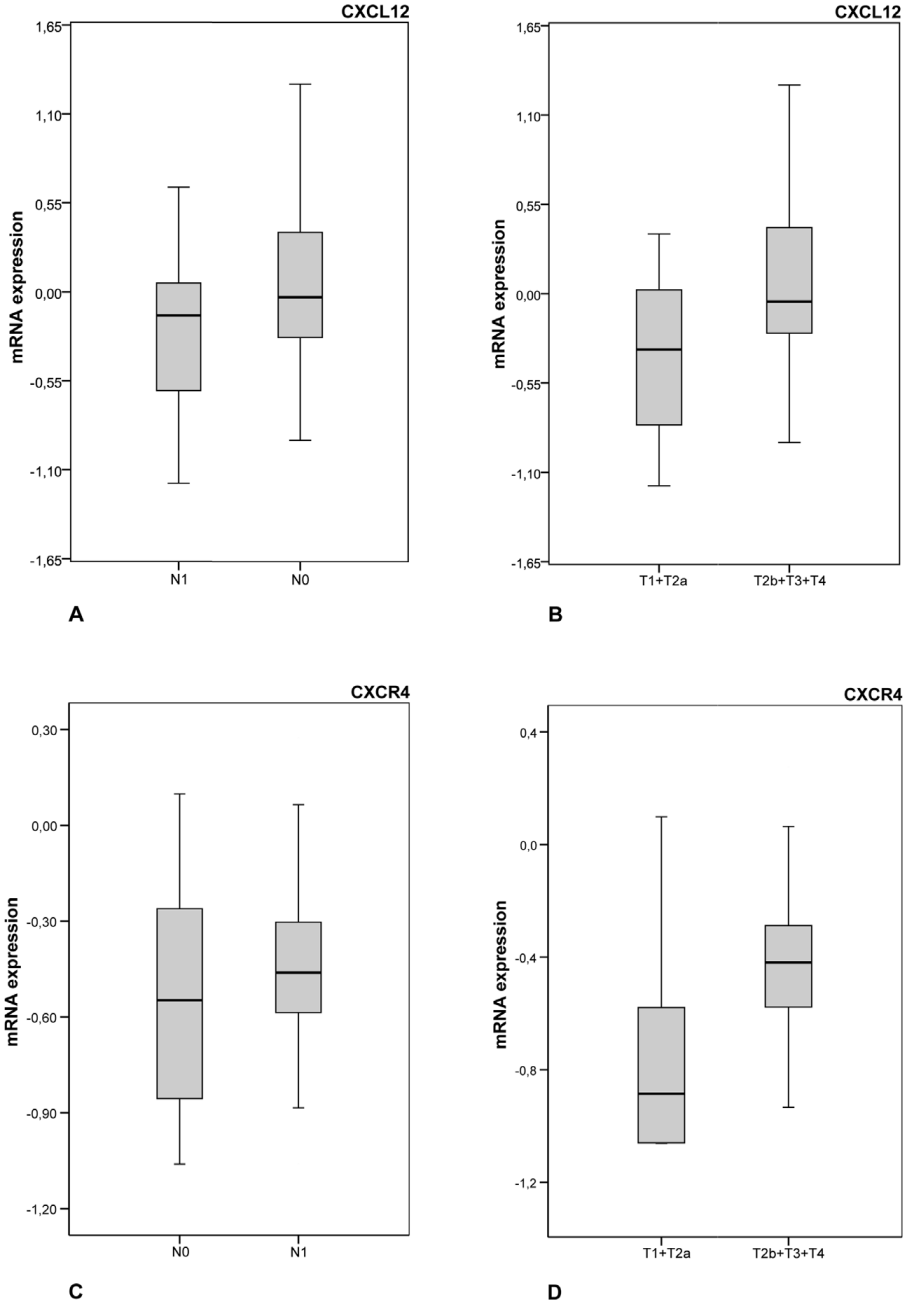
Transcription of CXCL12 and CXCR4: Boxplots depicting overall distribution of CXCL12 in (**a**) nodal negative versus nodal positive gastric carcinomas and in (**b**) pT1/T2a versus pT2b/T3/T4 gastric carcinomas. Overall distribution of CXCR4 in (**c**) nodal negative versus nodal positive gastric carcinomas and (**d**) pT1/T2a versus pT2b/T3/T4 gastric carcinomas. Box boundaries: 25^th^ and 75^th^ percentiles; solid line: median; whiskers: 10^th^ and 90^th^ percentiles.

CXCL12 expression was significantly increased in gastric carcinoma compared with non-neoplastic mucosa (p = 0.033). Confirming the array data, CXCL12 expression was also up-regulated in nodal positive gastric carcinoma compared with nodal negative cases. However, this difference did not reach statistical significance (p = 0.132; **figure 1a**). Concerning local tumour growth, there was no significant difference in CXCL12 expression in pT1/T2 stage tumour versus pT3/T4 tumours. But interestingly, CXCL12 expression showed a significant association in pT1/pT2a versus pT2b/T3/T4 (p = 0.049; **figure 1b**).

There was neither a difference of CXCR4 expression in gastric carcinoma versus non-neoplastic tissue (p = 0.229) nor in nodal negative versus nodal positive gastric carcinoma (p = 0.22; **figure 1c**). Comparing CXCR4 expression with the local tumour extent, CXCR4-mRNA levels increased with the local tumour growth (p = 0.079; **figure 1d**).

### Translation of CXCL12 in gastric carcinoma, correlation with clinicopathological parameters and survival analyses

**Figure 2:**
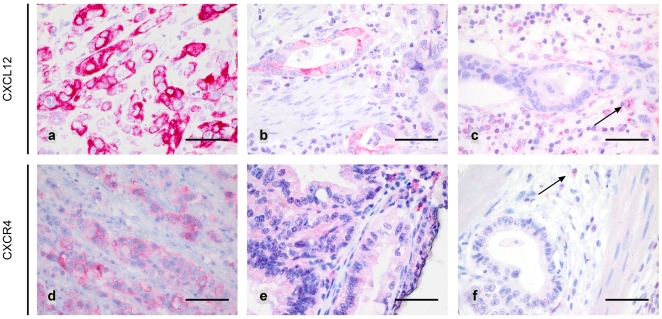
CXCL12 and CXCR4 expression in tumour cells: Gastric carcinoma samples revealing strong (**a**) and weak (**b**) CXCL12 immunoreactivity. Only few cases were CXCL12 negative (**c**). Note positive CXCL12 staining of blood vessels (arrow). Gastric carcinoma specimens showing a clear cytoplasmic and membranous CXCR4 immunoreactivity were sparse (**d**). Few samples revealed a weak (**e**) CXCR4 staining whereas most of the tumours lacked CXCR4 expression (**f**). Leukocytes served as internal positive control (arrows). Scale bar: a-f: 50 µm.

The translation and histoanatomical distribution of CXCL12 was subsequently studied by immunohistochemistry in 347 gastric carcinoma samples. In 291 cases, CXCL12 immunoreactivity was assessable. Tumour cells expressed CXCL12 in 244 of 291 (84%) samples. A strong cytoplasmic and membranous immunoreaction was observed in 143 (49%) cases and a weak staining in 101 (35%). 47 tumours (16%) lacked CXCL12-immunoreactivity. All tumour samples showed a distinct CXCL12 positivity of the vascular endothelial cells, which served as an internal positive control **(** **figure 2a-c** **)**. The statistical analyses showed a significant correlation between the expression of CXCL12 in tumour cells and distant metastases (p = 0.043) as well as tumour grade (p = 0.0064). All other clinicopathological parameters showed no association with CXCL12 expression in tumour cells **(** **table 1** **)**. When dividing the cohort into two groups, i.e. intestinal type and diffuse type gastric carcinoma, no correlation was found between CXCL12 expression and any clinicopathological parameter of either group (data not shown). The CXCL12 expression in tumour cells had no impact on patient survival (entire group: p = 0.830; intestinal type carcinoma: p = 0.766; diffuse type carcinoma: p = 0.817).

**Table 1:** Correlation of CXCL12-expression in tumour cells with clinicopathological patient characteristics.

Gastric carcinoma	Patients	CXCL12 immunoreactivity		
		0	1	P
Total	347			
**Age, years**				
≤65, n (%)	138	20 (14)	118 (86)	ns (p = 0.525)
>65, n (%)	153	27 (18)	126 (82)	
**Gender**				
men, n (%)	187	32 (17)	155 (83)	ns (p = 0.621)
women, n (%)	103	15 (15)	88 (85)	
**T category**				
pT1/pT2a, n (%)	48	8 (17)	40 (83)	ns (p = 1.0)
pT2b/pT3/pT4, n (%)	241	39 (16)	202 (84)	
pT1/pT2, n (%)	151	24 (16)	127 (84)	ns (p = 1.0)
pT3/pT4, n (%)	140	23 (16)	117 (84)	
**Lymph nodes**				
no metastases (%)	74	8 (11)	66 (89)	ns (p = 0.20)
Metastases (%)	215	39 (18)	176 (82)	
pN0, n (%)	74	8 (11)	66 (89)	ns (p = 0.22)
pN1, n (%)	103	16 (16)	87 (84)	
pN2, n (%)	78	14 (18)	64 (82)	
pN3, n (%)	34	9 (26)	25 (74)	
**M category**				
pM0, n (%)	257	37 (14)	220 (86)	p = 0.043
pM1, n (%)	34	10 (29)	24 (71)	
**Grade**				
G1/G2, n (%)	77	5 (6)	72 (94)	p = 0.0064
G3/G4, n (%)	214	42 (20)	172 (80)	
**UICC**				
I, n (%)	57	10 (18)	47 (82)	ns (p = 0.262)
II, n (%)	73	7 (10)	66 (90)	
III, n (%)	95	14 (15)	79 (85)	
IV, n (%)	68	16 (24)	52 (76)	

### Translation of CXCR4 in gastric carcinoma, correlation with clinicopathological parameters and survival analyses

**Figure 3:**
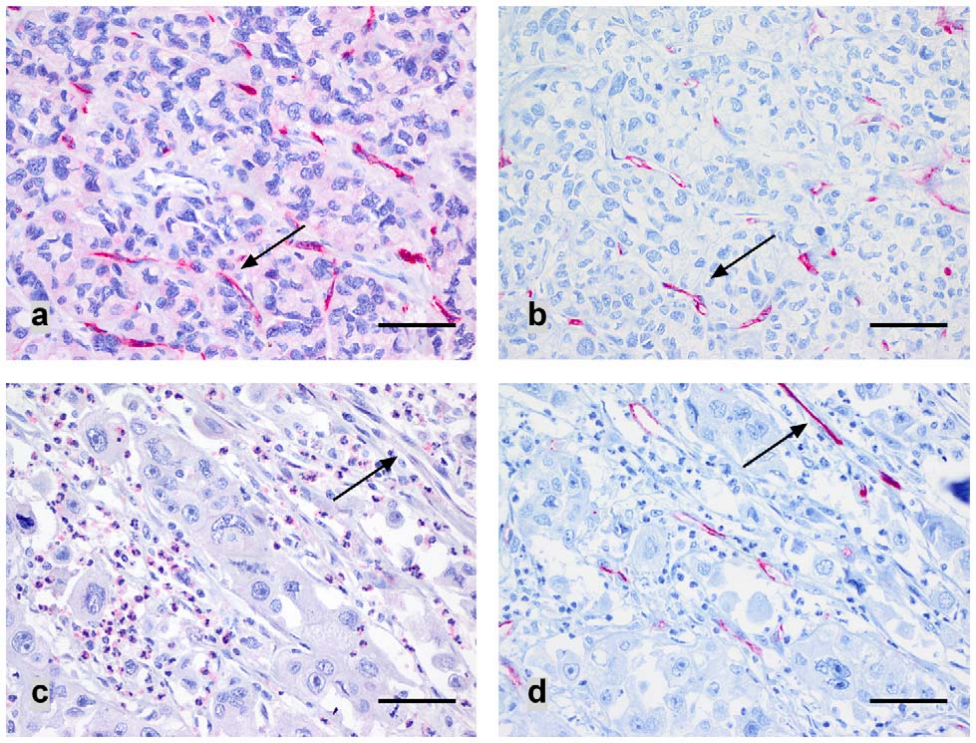
CXCR4 expression in tumour microvessels: Gastric carcinoma samples showing strong vascular CXCR4 immunoreactivity or lacking CXCR4 expression (**a,c**), indicated by arrows. Vascular structures were confirmed by a CD34 immunostaining (**b, d**). Scale bar: a-d: 50 µm.

Translation of CXCR4 was also studied by immunohistochemistry. Immunoreactivity in tumour cells was assessable in 293 tumour samples, of which only 6 (2%) showed an unequivocal membranous staining (category 2+; **figure 2d**). Most of the CXCR4 positive tumour specimens only revealed a faint cytoplasmic CXCR4 immunoreactivity (category 1+, 44 cases, 15%; **figure 2e**). Nuclear CXCR4 immunoreactivity was not observed in any case and any cell type. An overall of 83% (243 of 293) of the gastric carcinomas were immunonegative for CXCR4, most of them showing concomitant CXCR4 positive leucocytes as an internal positive control (**figure 2f**). Interestingly, as previously observed in colorectal carcinomas, 86 of 293 gastric carcinomas (29%) showed CXCR4 positive microvessels in the tumour stroma with a strong CXCR4 immunoreactivity of endothelial cells (**figure 3a**). The vascular nature of these delicate structures was confirmed by a CD34 immunostaining (**figure 3b**).

When correlating CXCR4 expression in tumour cells with various clinicopathological parameters, CXCR4 expression was significantly associated with higher local tumour extent (T-status; p = 0.030). However, no further associations of tumoral CXCR4 expression and other clinicopathological variables were found **(** **table 2** **)**. When analyzing the subgroup of intestinal type gastric carcinomas, no associations were found with any clinicopathological parameter (data not shown).

**Table 2:** Correlation of CXCR4 expression in tumour cells with clinicopathological patient characteristics.

Gastric carcinoma	Patients	CXCR4 immunoreactivity of tumour cells		
		0	1	P
Total	347			
**Age, years**				
≤65, n (%)	142	119 (84)	23 (16)	ns (p = 0.757)
>65, n (%)	151	124 (82)	27 (18)	
**Gender**				
men, n (%)	187	154 (82)	33 (18)	ns (p = 0.629)
women, n (%)	105	89 (85)	16 (15)	
**T category**				
pT1/pT2a, n (%)	52	43 (83)	9 (17)	ns (p = 1.0)
pT2b/pT3/pT4, n (%)	239	199 (83)	40 (17)	
pT1/pT2, n (%)	153	134 (88)	19 (12)	p = 0.030
pT3/pT4, n (%)	140	109 (78)	31 (22)	
**Lymph nodes**				
no metastases (%)	74	60 (86)	14 (14)	ns (p = 0.721)
Metastases (%)	217	181 (83)	36 (17)	
pN0, n (%)	74	60 (86)	14 (14)	ns (p = 0.83)
pN1, n (%)	104	89 (86)	15 (14)	
pN2, n (%)	79	64 (81)	15 (19)	
pN3, n (%)	34	28 (82)	6 (18)	
**M category**				
pM0, n (%)	258	215 (83)	43 (17)	ns (p = 0.633)
pM1, n (%)	35	28 (80)	7 (20)	
**Grade**				
G1/G2, n (%)	78	69 (88)	9 (12)	ns (p = 0.163)
G3/G4, n (%)	215	174 (81)	41 (19)	
**UICC**				
I, n (%)	59	50 (85)	9 (15)	ns (p = 0.1413)
II, n (%)	73	66 (90)	7 (10)	
III, n (%)	91	70 (77)	21 (23)	
IV, n (%)	70	57 (81)	13 (19)	

Then we studied the correlation between CXCR4 expression in endothelial cells (vascular CXCR4 expression, vCXCR4) of tumour microvessels and various clinicopathological parameters. Interestingly, the expression of CXCR4 in microvessels correlated highly significantly with the local tumour growth (T-category; p = 0.0001) as well as with the UICC-tumour stage (p = 0.0059). Even in the subgroups of intestinal type and diffuse type gastric carcinoma, vCXCR4 expression was significantly associated with local tumour extent (intestinal type: p = 0.004; diffuse type: p = 0.030) and UICC-tumour stage (intestinal type: p = 0.020). Furthermore vCXCR4 expression was significantly associated with patient age (p = 0.0148) in the entire group (**table 3**).

**Table 3:** Correlation of vascular CXCR4 expression with clinicopathological patient characteristics.

Gastric carcinoma	Patients	Vascular CXCR4 immunoreactivity		
		0	1	P
Total	347			
**Age, years**				
≤65, n (%)	142	110 (77)	32 (23)	p = 0.0148
>65, n (%)	151	97 (64)	54 (36)	
**Gender**				
men, n (%)	187	132 (71)	55 (29)	ns (p = 1.0)
women, n (%)	105	74 (70)	31 (30)	
**T category**				
pT1/pT2a, n (%)	52	48 (92)	4 (8)	p = 0.0001
pT2b/pT3/pT4, n (%)	239	158 (66)	81 (34)	
pT1/pT2, n (%)	154	124 (81)	30 (19)	p = 0.0001
pT3/pT4, n (%)	139	83 (60)	56 (40)	
**Lymph nodes**				
no metastases (%)	74	55 (74)	19 (26)	ns (p = 0.463)
Metastases (%)	217	151 (70)	66 (30)	
pN0, n (%)	74	55 (74)	19 (26)	ns (p = 0.689)
pN1, n (%)	105	75 (71)	30 (29)	
pN2, n (%)	79	52 (66)	27 (34)	
pN3, n (%)	33	24 (73)	9 (27)	
**M category**				
pM0, n (%)	259	185 (71)	74 (29)	ns (p = 0.427)
pM1, n (%)	34	22 (65)	12 (35)	
**Grade**				
G1/G2, n (%)	78	54 (69)	24 (31)	ns (p = 0.773)
G3/G4, n (%)	215	153 (71)	62 (29)	
**UICC**				
I, n (%)	58	52 (90)	6 (10)	p = 0.0059
II, n (%)	74	51 (69)	23 (31)	
III, n (%)	91	56 (62)	35 (38)	
IV, n (%)	69	48 (70)	21 (30)	

Survival analysis showed that CXCR4 expression in tumour cells of gastric carcinoma as well as in tumour microvessels had no impact on survival.

### Concomitant CXCL12/vCXCR4 expression in gastric carcinoma

Since the CXCL12-CXCR4 axis has been shown to be involved in tumour progression [17]–[19], we investigated the correlation of the concomitant expression of CXCL12 and vCXCR4 with clinicopathological parameters. Indeed, CXCL12-vCXCR4 positive tumours were associated with higher local tumour extent (p = 0.0014) and higher UICC stages (p = 0.017). However, it had no impact on patient survival, even in the subgroups of intestinal and diffuse type gastric carcinoma.

### Expression pattern of CXCL12 and CXCR4 in matching lymph node metastases

The CXCL12-CXCR4 axis has been reported to be involved in metastatic processes in various tumour entities. Therefore we examined CXCL12 and CXCR4 immunoreactivity in a subset of 61 matching lymph node metastases. The CXCL12 expression pattern was available for 46 metastases. Overall, 4 lymph node metastases were CXCL12 negative like their corresponding primary tumour. 40 lymph node metastases showed a clear CXCL12 positivity according to the primary tumour. The staining intensity was very heterogeneous showing strongly positive tumour cells adjacent to faintly stained tumour cell clusters. However, two metastases revealed CXCL12 immunoreactivity although no CXCL12 expression has been detected in the primary tumour.

CXCR4 immunoreactivity was assessed in 54 lymph node metastases. Interestingly, none of them showed any CXCR4 expression. Even those tumours (n = 6), showing a faint CXCR4 positivity in the primary tumour, lacked CXCR4 expression in the corresponding lymph node metastases. However, all lymph node metastases revealed clearly CXCR4 positive lymphocytes, which served as internal positive control. Interestingly, in three cases intratumoural CXCR4 positive microvessels were detectable.

## Discussion

G-protein-coupled receptors represent the largest family of transmembrane receptors. Five percent of all human genes code for more than 800 different GPCRs and approximately 80 different ligands were identified until now [20]. GPCRs are the most common pharmacological targets in medicine, i.e. almost 30% of all drugs are directed against GPCRs. Evidence is increasing that GPCRs may also be promising targets for cancer therapy. In this study we aimed to find GPCRs that are differentially expressed in node positive gastric cancers and hence may be considered as future targets for gastric cancer treatment. We found 52 GPCRs and GPCR-related genes that are up- or down-regulated in node positive primary gastric cancer tissue compared with node negative cancer. Several of the GPCRs were formerly shown to be involved in tumour biology, such as AT1R, EDG2, DARC, and FPR1 [10], [11], [14], [15]. Most interestingly, our list also included LGR5 [16], which was recently shown to be a stem cell marker in the small intestine and colon. Furthermore, specific loss of Apc in LGR5-positive cells results in progressively growing neoplasias. Thus, *in silico* validation of our data supported the hypothesis that GPCRs are involved in the tumour biology of gastric cancer.

Our subsequent validation studies using a group of independent patients showed that the GPCR-ligand CXCL12 is expressed in tumour cells of the majority of gastric carcinomas. Furthermore, CXCL12 expression is negatively associated with distant metastases and tumour grade. To the contrary, only 17% of gastric carcinomas showed CXCR4 immunopositive tumour cells, which was associated with higher local tumour extent. Interestingly, about one third of the gastric carcinomas showed CXCR4 positive tumour microvessels. Vascular CXCR4 expression was significantly associated with higher local tumour extent as well as higher UICC-stages. When expressing both, CXCL12 in tumour cells and CXCR4 in tumour microvessels, these tumours also were significantly associated with higher T- and UICC-stages, supporting the role of the CXCL12-CXCR4 axis in neoangiogenesis of gastric cancer.

Among the GPCRs, the chemokine system contributes significantly to tumour progression through modulation of the local inflammatory reaction, tumour cell proliferation, migration and survival as well as neoangiogenesis [4]. The chemokine receptor CXCR4 initially was found to regulate the homing of lymphocytes in inflammation and represents a co-receptor for the human immunodeficiency virus (HIV) [21]. Physiologically, the CXCL12-CXCR4 axis is involved in migration of embryonic cells of the central nervous system, bone marrow and heart [22], [23]. It plays a critical role in metastatic processes as shown for breast [24], [25], ovarian [26] and prostate cancer [27] and CXCL12 is highly expressed in organs which are frequent sites of distant metastases like lung, liver and lymph nodes [28]. In gastric carcinoma, data concerning the CXCL12-CXCR4 pathway are sparse [29]. There is some evidence that CXCR4 expressing gastric carcinomas more likely develop a peritoneal spread of the tumour, and malignant ascites contains high concentrations of CXCL12 [30].

Comparing our gene array data with those obtained by RT-PCR and immunohistochemistry, it was interesting to note that the differential expression of CXCL12 in node positive gastric carcinoma was confirmed on the transcriptional but not on the translational level. Here, the immunohistochemical detection of CXCL12 in tumour cells correlated only with distant metastases and tumour grade but not with nodal spread. However, CXCL12 was found not only in tumour cells, but also in endothelial and stromal cells [31], and overall expression in the entire tissue compartment is more difficult to assess by immunohistochemistry. To the contrary, our transcriptional studies used tissue homogenates which integrate all CXCL12 expressing cells of the tumour tissue. Nevertheless, it has to be kept in mind that gene transcription does not always correlate with mRNA translation and that the tumour biological effect of CXCL12 may also depend on the presence and histoanatomical distribution of CXCR4. In support of this contention, we were able to show that concomitant expression of CXCL12 in tumour cells and CXCR4 in tumour microvessels correlated with local tumour growth and UICC-tumour stage.

It was reported that tumoural CXCR4 positivity significantly correlates with the development of peritoneal carcinomatosis [30]. Furthermore, strong CXCR4 expression was significantly associated with lymph node metastases, higher UICC stages and a reduced 5-year survival rate [32]. Our results appear to differ from these previous studies. When evaluating CXCR4 immunoreactivity in tumour cells, a low expression rate was observed. Only 17% of the tumour samples showed a mostly faint CXCR4 immunoreactivity. Furthermore, all 61 matching lymph node metastases lacked CXCR4 expression. This staining pattern may explain, why for example in intestinal type gastric carcinomas, CXCR4 expression only was significantly associated with the local tumour growth (T-category) but not with other clinicopathological factors as previously described. We used a thoroughly characterized CXCR4 antibody with a higher specificity than commercially available antibodies as shown by Fisher and colleagues [33]. This difference in specificity could serve for different staining patterns. For example, a nuclear CXCR4 expression, which was reported to be associated with lymph node metastases in colorectal cancer, was not seen in our series [34]. Furthermore, we never detected CXCR4 positivity in stromal cells as described in a study of Saigusa and colleagues [35]. To clarify, if the CXCR4-CXCL12 pathway ultimately contributes to generation of metastases in gastric carcinoma, especially lymph node metastases, further studies are needed.

About one third of the examined gastric carcinomas showed CXCR4 positive tumour- surrounding microvessels. Tumour cells require adequate supply of oxygen and nutrients to maintain survival. Even with genetic abnormalities that dysregulate growth and survival of individual cells, tumours cannot enlarge beyond 1–2 mm^3^ without vascularisation and hypoxia-induced cell death occurs. It has been shown that CXCR4 is expressed by endothelial cells and stimulation of CXCR4 by CXCL12 has a proangiogenic effect [36]. Furthermore, CXCR4 is a hypoxia inducible gene, regulated by the hypoxia-induced factor 1α (HIF1α). When oxygen is scarce like in rapidly growing tumours, HIF1α enhances the expression CXCR4 [37]. The increased expression of CXCR4 in endothelial cells observed in our tumour collective might be part of an integrated hypoxic response of the growing tumour that allows for the generation of new blood vessels. In our study group, vascular CXCR4 expression correlated significantly with the extent of local tumour growth. In 8% of T1/pT2a tumours (4 of 52) and in 34% of T2b/T3/pT4 tumours (81 of 239) CXCR4 positive microvessels were detectable. T2b/T3/T4-stage gastric carcinomas more likely harbour a hypoxic microenvironment than T1/T2a-stage tumours and thereby might induce CXCR4 gene expression and angiogenesis. Detection of CXCR4 positive microvessels in large lymph node metastases (>9 mm in diameter) corroborated these observations. Furthermore, we have previously shown comparable results in colorectal carcinoma [38]. Additionally, as shown in glioblastoma multiforme [39], tumour samples revealing both, CXCL12 positive tumour cells and CXCR4 positive microvessels were highly significantly associated with high local tumour extent and high UICC stages, further supporting the significance of a functional CXCL12-CXCR4 axis in gastric cancer biology.

In summary, we show that GPCRs are differentially expressed in gastric cancer tissue and may contribute to the tumour biology: tumours expressing both, CXCL12 in tumour cells and CXCR4 in tumour surrounding microvessels, show a highly significant association with local tumour growth and UICC stages. These results, together with our previous data on colorectal carcinoma, substantiate the role of the CXCL12-CXCR4 axis in tumour-neoangiogenesis in gastrointestinal tumours. The CXCL12-CXCR4 pathway might be novel promising antiangiogenic target for the treatment of gastric carcinomas.

## Materials and Methods

### Tumour samples

Tissue samples of gastric cancer were obtained surgically at the Charité University Hospital Berlin (1995–2008). Fresh frozen tissue of 6 cases of intestinal type gastric carcinoma was used for GeneChip analysis (nodal negative: 3 patients; nodal positive: 3 patients; female-male-ratio: 1∶2). An independent series of paired cancerous and tumor-adjacent normal tissues from 37 intestinal type gastric carcinomas were examined by real-time RT-PCR (nodal negative: 12 patients; nodal positive: 25 patients; female-male-ratio: ∼1∶1, for detailed patient characteristics see table S3). For immunohistochemical analyses, a patient cohort of 347 consecutive patients with gastric cancer was examined, comprising 194 intestinal type and 122 diffuse type carcinomas according to the Laurén classification. 31 samples showed other histological subtypes (i.e. mucinous, tubular, undifferentiated). Briefly, the cohort consisted of 220 men and 127 women. The mean age of the patients at the time of diagnosis was 64 years. Survival data was available from 196 of these patients. Follow-up data for the other patients was missing because these patients were not resident near the hospital and were lost to follow-up. Out of 196 patients, 124 died during follow-up. Median follow-up for those patients still alive at the endpoint of analysis was 33 months. Of 61 patients, tissue of matching lymph node metastases was available (27 intestinal type, 26 diffuse type, 8 other histological subtypes). Only patients with histologically confirmed gastric cancer and adequate tissue available were included. Patients with neoadjuvantly treated gastric carcinoma or other known malignancies were excluded from the study. This project was approved by the local ethics committee (ref. number EA1/06/2004).

### GeneChip analysis

Total RNA was isolated with phenol-chloroform using the mirVana™ miRNA Isolation Kit (Ambion, Austin, USA). Contaminating DNA was removed by DNase treatment (Turbo DNAfree kit; Ambion, Austin, USA) at 37°C for 30 min. We used the GeneChip® Human Genome U133 Plus 2.0 arrays (Affymetrix, Santa Clara, CA, USA) according to the manufacturers protocol to analyze mRNA expression levels. Affymetrix GeneChip® Operating Software (GCOS 1.4) automates the control of GeneChip® Fluidics Stations and GeneChip® Scanner 3000.

### Bioinformatics

Raw data were analyzed with the Affymetrix GeneChip Operating Software (GCOS 1.4). The detection p-value of a transcript determines the detection call, which indicates whether the transcript is reliably detected (p<0.05; present) or not detected (absent). To enable the comparison between chips the data were scaled to a global intensity of 500. The Data Mining Tool 3.0 (Affymetrix) and GeneSpring software package 7.2 (Silicon Genetics, Redwood City, CA) were used to average results from different samples and perform statistical analysis to compare between gastric cancer with (N1) and without (N0) lymph node metastases. The data of six arrays were normalized to account for variability in hybridization for probe pairs and other hybridization artefacts. The normalization consists of the following three steps: first, data transformation (set measurements less than 300.0 to 300.0); second, per chip (normalize each chip to the 50th percentile of the measurements taken from that chip); and third, per gene (normalize each gene to the median of the measurements for that gene). The fold change was calculated for each gene as the arithmetic mean of the normalized expression values of N1 divided by the arithmetic mean of the normalized expression values of N0. Raw data from microarray experiments were uploaded to the Gene Expression Omnibus Database (http://www.ncbi.nlm.nih.gov/geo/query/acc.cgi?token=pzmjlcoscakugfc&acc=GSE17187).

### Real-time reverse transcriptase polymerase chain reaction

For cDNA synthesis, 2 µg of total RNA was reverse transcribed using the Omniscript RT Kit (Qiagen). The gene-specific primers were designed by the BioTeZ Berlin-Buch GmbH (Berlin, Germany). Primer sequences were as follows: **CXCR4** 5′ CAG CAG GTA GCA AAG TGA CG, 3′ CAG GGT TCC TTC ATG GAG TC; **CXCL12** 5′ CGA TTC TTC GAA AGC CAT GT, 3′CAC TTG TCT GTT GTT GTT CTT CAG; **beta2-microglobulin** 5′ ACC CCC ACT GAA AAA GAT GA, 3′ ATC TTC AAA CCT CCA TGA TG. Real-time reverse-transcriptase polymerase chain reaction (Real-time RT-PCR) was carried out using the QuantiTect™ SYBR® Green PCR Kit (Qiagen) and the LightCycler System (Roche Diagnostics, Mannheim, Germany). The comparative C_t_ values were normalized to that of the housekeeping gene beta2-microglobulin. No template controls (no cDNA in PCR) were run for each gene to detect unspecific or genomic amplification and primer dimerization.

### Histology

For histological analyses, tissue samples were fixed in 10% neutralized formalin and embedded in paraffin. Deparaffinized sections were stained using hematoxylin and eosin. Gastric carcinoma was classified according to the WHO classification [1]. The pTNM stage was determined according to the UICC guidelines.

### Tissue microarray construction

Formalin-fixed and paraffin-embedded tissue samples were used to generate tissue microarrays as described previously [40], [41]. Briefly, three to six morphologically representative regions of the paraffin ‘donor’ blocks were chosen. Tissue cylinders were punched from these areas and precisely arrayed into a new ‘recipient’ paraffin block using a customer built instrument (Beecher Instruments, Silver Spring, MD, USA). A minimum of three tissue cylinders of 0.6 mm diameter were punched from each sample. After completing the block construction, four micrometer sections of the resulting tumour tissue microarray block were cut for further analysis.

### Immunohistochemistry

Immunostaining was carried out with an anti-CXCR4-antiserum (dilution 1∶100; rabbit polyclonal antiserum;[38]) and an anti-CXCL12-antibody (dilution 1∶100; mouse monoclonal IgG1; R&D Systems, Minneapolis, MN, USA). Following antigen retrieval (sodium-citrate, 4×5 min, 600 W, microwave oven), incubation with the primary antibodies was performed in a moist chamber at 4°C overnight. Slides were washed between steps with Tris-buffered saline. Immunoreactions were visualized with the Super Sensitive Link Label Detection System (BioGenex Laboratories, San Ramon, CA, USA) combined with the SIGMA*FAST™* kit (Sigma-Aldrich, St. Louis, MO, USA). The specimens were counterstained with hematoxylin. Immunostaining with an anti-CD34-antibody (dilution 1∶100; mouse monoclonal IgG1, kappa; DAKO, Carpinteria, CA, USA) used the Ventana Benchmark XT automated staining system (Ventana Medical Systems, Tucson, AZ, USA). Omission of primary antibodies served as negative controls. Normal human adrenal gland tissue served as positive control for CXCR4, normal human tonsil tissue for CXCL12. The CXCR4 immunoreactivity in tumour cells was categorized as absent (0), faint cytoplasmic staining (1+), clear cytoplasmic and/or clear membranous staining (2+). CXCR4 expression in tumour microvessels was recorded as positive or negative. CXCL12 immunoreactivity in primary tumours was scored as absent (0), weak cytoplasmic (1) or strong cytoplasmic (2). CXCL12 expression in lymph node metastases was scored as positive or negative. All samples were evaluated by one pathologist (BI). When evaluating the sections, discrepancy in sample number was related to tissue loss during the transfer of the TMA sections onto the slides.

### Statistical analyses

For statistical analyses 1+ and 2+ tumour samples were considered as CXCR4 positive (1) whereas tumours with lack of immunoreactivity were scored as negative (0). Vascular CXCR4 expression always showed a strong signal and was recorded as positive (1) or negative (0). 1+ and 2+ CXCL12 immunoreactivity was scored as positive, whereas tumour samples lacking CXCL12 immunoreactivity were scored as negative. Significance of correlations between protein expression (CXCR4 and CXCL12) and clinicopathological parameters was assessed by Fisher's exact test for 2×2 tables and by the chi squared test for larger tables. Survival curves were fitted with the Kaplan-Meier method. Differences in survival were assessed by the log rank test.

Real-time RT-PCR data was logarithmized to obtain approximately normally distributed data. Results were evaluated with an unpaired two-sided t-test. P-values<0.05 were considered as statistically significant. Statistical analyses were performed using the SPSS 17 statistical package (SPSS Inc., Chicago, IL, USA) or the GraphPad Prism statistical software (GraphPad Software, Inc. La Jolla, CA, USA).

## Supporting Information

Table S1 Differentially expressed genes in the primary tumours of node- negative (N0) vs. node-positive (N1) intestinal type primary gastric carcinomas based on microarray analysis (fold change factor >1.7).
(0.32 MB DOC)

Table S2 Differentially expressed GPCRs and GPCR-related genes in the primary tumors of node-negative (N0) vs. node-positive (N1) intestinal type primary gastric carcinomas based on microarray analysis (fold change factor >1.5).
(0.11 MB DOC)

Table S3 Patient characteristics of RT-PCR validation sample set.
(0.06 MB DOC)
