# Supplementary material for: Vascular CXCR4 Expression – a Novel Antiangiogenic Target in Gastric Cancer?
Source: PLoS One. 2010 Apr 8;5(4):e10087. doi: 10.1371/journal.pone.0010087 (PMC2851611; doi:10.1371/journal.pone.0010087)
Supplement: Table S1 — Differentially expressed genes in the primary tumours of node- negative (N0) vs. node-positive (N1) intestinal type primary gastric carcinomas based on microarray analysis (fold change factor >1.7). (0.32 MB DOC) [file pone.0010087.s001.doc]

**Table S1:** Differentially expressed genes in the primary tumours of node- negative (N0) vs. node-positive (N1) intestinal type primary gastric carcinomas based on microarray analysis (fold change factor >1.7).

| **N0 vs N1 down** |  |  |
| --- | --- | --- |
| **Probe Set ID** | **Fold Change** | **Gene Title** |
| 212140_at | 0.769 | KIAA0648 protein |
| 209838_at | 0.769 | thyroid receptor interacting protein 15 |
| 201454_s_at | 0.769 | aminopeptidase puromycin sensitive |
| 226156_at | 0.768 | v-akt murine thymoma viral oncogene homolog 2 |
| 226927_at | 0.768 | CDNA clone IMAGE:5269587. partial cds |
| 225718_at | 0.768 | KIAA1715 |
| 226501_at | 0.767 | 601661316R1 NIH_MGC_72 Homo sapiens cDNA clone IMAGE:3916274 3'. mRNA sequence. |
| 207541_s_at | 0.767 | polymyositis/scleroderma autoantigen 2. 100kDa |
| 214753_at | 0.767 | xc26c06.x1 NCI_CGAP_Co18 Homo sapiens cDNA clone IMAGE:2585386 3' similar to contains element OFR repetitive element ;. mRNA sequence. |
| 212131_at | 0.766 | chromosome 19 open reading frame 13 |
| 204243_at | 0.766 | rearranged L-myc fusion sequence |
| 203374_s_at | 0.766 | tripeptidyl peptidase II |
| 201757_at | 0.765 | NADH dehydrogenase (ubiquinone) Fe-S protein 5. 15kDa (NADH-coenzyme Q reductase) |
| 227837_at | 0.765 | hypothetical protein FLJ20309 |
| 202090_s_at | 0.765 | ubiquinol-cytochrome c reductase (6.4kD) subunit |
| 204193_at | 0.763 | carnitine palmitoyltransferase 1B (muscle) |
| 243280_at | 0.763 | Transcribed sequences |
| 1569180_at | 0.762 | Clone IMAGE:4250282. mRNA |
| 228256_s_at | 0.761 | TIGA1 |
| 223331_s_at | 0.76 | DEAD (Asp-Glu-Ala-Asp) box polypeptide 20 |
| 227982_at | 0.76 | soluble liver antigen/liver pancreas antigen |
| 1557737_s_at | 0.758 | natural killer-tumor recognition sequence |
| 215031_x_at | 0.757 | ring finger protein 126 |
| 226635_at | 0.757 | 602322848F1 NIH_MGC_89 Homo sapiens cDNA clone IMAGE:4426211 5'. mRNA sequence. |
| 217893_s_at | 0.757 | hypothetical protein FLJ12666 |
| 222473_s_at | 0.756 | erbb2 interacting protein |
| 230379_x_at | 0.755 | hypothetical protein PRO1853 |
| 212330_at | 0.755 | transcription factor Dp-1 |
| 213000_at | 0.755 | Homo sapiens genomic DNA. chromosome 21q22.2. BAC clone:KB739C11. CBR1-HLCS region. |
| 235767_x_at | 0.755 | likely ortholog of mouse phosphorylated adaptor for RNA export |
| 223335_at | 0.754 | hypothetical protein LOC51249 |
| 47069_at | 0.754 | Rho GTPase activating protein 8 |
| 227998_at | 0.753 | S100 calcium binding protein A16 |
| 226170_at | 0.753 | eyes absent homolog 3 (Drosophila) |
| 204241_at | 0.753 | acyl-Coenzyme A oxidase 3. pristanoyl |
| 201795_at | 0.753 | lamin B receptor |
| 218068_s_at | 0.752 | hypothetical protein FLJ22301 |
| 214728_x_at | 0.752 | SWI/SNF related. matrix associated. actin dependent regulator of chromatin. subfamily a. member 4 |
| 219194_at | 0.752 | sema domain. immunoglobulin domain (Ig). transmembrane domain (TM) and short cytoplasmic domain. (semaphorin) 4G |
| 221537_at | 0.751 | hypothetical protein DKFZp564A176 |
| AFFX-BioDn-3_at | 0.751 |  |
| 200854_at | 0.751 | nuclear receptor co-repressor 1 |
| 221813_at | 0.75 | KIAA1332 protein |
| 227663_at | 0.749 | Transcribed sequences |
| 202331_at | 0.749 | branched chain keto acid dehydrogenase E1. alpha polypeptide (maple syrup urine disease) |
| 208616_s_at | 0.749 | protein tyrosine phosphatase type IVA. member 2 |
| 224635_s_at | 0.748 | baculoviral IAP repeat-containing 6 (apollon) |
| 239557_at | 0.748 | Transcribed sequence with weak similarity to protein ref:NP_060312.1 (H.sapiens) hypothetical protein FLJ20489 [Homo sapiens] |
| 223724_s_at | 0.747 | Similar to Cohesin subunit SA-3 (Stromal antigen 3) (SCC3 homolog 3) (LOC392725). mRNA |
| 204593_s_at | 0.747 | hypothetical protein FLJ20232 |
| 227604_at | 0.746 | family with sequence similarity 11. member B |
| 214170_x_at | 0.746 | fumarate hydratase |
| 202573_at | 0.745 | casein kinase 1. gamma 2 |
| 218152_at | 0.745 | high-mobility group 20A |
| 225740_x_at | 0.744 | Mdm4. transformed 3T3 cell double minute 4. p53 binding protein (mouse) |
| 211584_s_at | 0.744 | nuclear protein. ataxia-telangiectasia locus |
| 1557432_at | 0.743 | Transcribed sequences |
| 225543_at | 0.743 | general transcription factor IIIC. polypeptide 4. 90kDa |
| 212375_at | 0.743 | E1A binding protein p400 |
| 235327_x_at | 0.742 | UBX domain containing 4 |
| 212744_at | 0.742 | Bardet-Biedl syndrome 4 |
| 232469_x_at | 0.742 | single stranded DNA binding protein 3 |
| 203093_s_at | 0.741 | translocase of inner mitochondrial membrane 44 homolog (yeast) |
| 219145_at | 0.741 | synonyms: LEC2. CIRL1. FLJ11939. KIAA0821; lectomedin-2; go_component: integral to membrane [goid 0016021] [evidence IEA]; go_function: sugar binding [goid 0005529] [evidence IEA]; go_function: G-protein coupled receptor activity [goid 0004930] [evidence IEA]; go_function: receptor activity [goid 0004872] [evidence IEA]; go_function: protein prenyltransferase activity [goid 0008318] [evidence IEA]; go_function: latrotoxin receptor activity [goid 0016524] [evidence IEA]; go_process: neuropeptide signaling pathway [goid 0007218] [evidence IEA]; go_process: protein amino acid prenylation [goid 0018346] [evidence IEA]; Homo sapiens latrophilin 1 (LPHN1). mRNA. |
| 224486_s_at | 0.74 | hypothetical protein HH114 |
| 222104_x_at | 0.739 | general transcription factor IIH. polypeptide 3. 34kDa |
| 211947_s_at | 0.739 | HBxAg transactivated protein 2 |
| 221516_s_at | 0.738 | hypothetical protein FLJ20232 |
| 212701_at | 0.737 | talin 2 |
| 227538_at | 0.735 | cofactor required for Sp1 transcriptional activation. subunit 7. 70kDa |
| 212388_at | 0.735 | ubiquitin specific protease 24 |
| 203921_at | 0.734 | carbohydrate (N-acetylglucosamine-6-O) sulfotransferase 2 |
| 200774_at | 0.734 | 601657412R1 NIH_MGC_68 Homo sapiens cDNA clone IMAGE:3875738 3'. mRNA sequence. |
| 202420_s_at | 0.734 | DEAH (Asp-Glu-Ala-His) box polypeptide 9 |
| 211733_x_at | 0.733 | sterol carrier protein 2 |
| 234923_at | 0.732 | GTPase activating RANGAP domain-like 1 |
| 203033_x_at | 0.732 | fumarate hydratase |
| 205248_at | 0.731 | chromosome 21 open reading frame 5 |
| 202161_at | 0.731 | protein kinase C-like 1 |
| 239771_at | 0.73 | 602441651F1 NIH_MGC_75 Homo sapiens cDNA clone IMAGE:4557325 5'. mRNA sequence. |
| 232412_at | 0.73 | CDNA: FLJ21037 fis. clone CAE10055 |
| 200797_s_at | 0.73 | qw03a03.x1 NCI_CGAP_Ut3 Homo sapiens cDNA clone IMAGE:1989964 3'. mRNA sequence. |
| 212748_at | 0.729 | megakaryoblastic leukemia (translocation) 1 |
| 207735_at | 0.729 | ring finger protein 125 |
| 203732_at | 0.727 | thyroid hormone receptor interactor 4 |
| 239143_x_at | 0.725 | ring finger protein 138 |
| 226889_at | 0.725 | WD repeat domain 35 |
| 227245_at | 0.725 | hypothetical protein FLJ13089 |
| 222366_at | 0.725 | Transcribed sequences |
| 226233_at | 0.725 | tubulin-specific chaperone e |
| 212628_at | 0.725 | protein kinase C-like 2 |
| 200972_at | 0.725 | transmembrane 4 superfamily member 8 |
| 202675_at | 0.724 | succinate dehydrogenase complex. subunit B. iron sulfur (Ip) |
| 1553528_a_at | 0.723 | TAF5 RNA polymerase II. TATA box binding protein (TBP)-associated factor. 100kDa |
| 206095_s_at | 0.723 | FUS interacting protein (serine-arginine rich) 1 |
| 221666_s_at | 0.723 | apoptosis-associated speck-like protein containing a CARD |
| 219168_s_at | 0.721 | Rho GTPase activating protein 8 |
| 208956_x_at | 0.721 | dUTP pyrophosphatase |
| 231850_x_at | 0.72 | KIAA1712 |
| 209195_s_at | 0.72 | adenylate cyclase 6 |
| 218763_at | 0.72 | syntaxin 18 |
| 232975_at | 0.719 | CDNA FLJ13272 fis. clone OVARC1001004 |
| 241792_x_at | 0.718 | yy29e02.s1 Soares melanocyte 2NbHM Homo sapiens cDNA clone IMAGE:272666 3'. mRNA sequence. |
| 204387_x_at | 0.718 | mitochondrial ribosomal protein 63 |
| 212310_at | 0.718 | C219-reactive peptide |
| 200074_s_at | 0.716 | ribosomal protein L14 |
| 224755_at | 0.715 | T84 colon carcinoma cell IL-1beta regulated HSCC1 mRNA. partial sequence |
| 212200_at | 0.715 | KIAA0692 protein |
| 225148_at | 0.715 | hypothetical protein MGC52010 |
| 224993_at | 0.715 | Clone IMAGE:4441633. mRNA |
| 204520_x_at | 0.713 | bromodomain containing 1 |
| 1554595_at | 0.711 | symplekin |
| 233940_at | 0.71 | Homo sapiens cDNA FLJ12739 fis. clone NT2RP2000498. |
| 230742_at | 0.71 | ny88h08.s1 NCI_CGAP_GCB1 Homo sapiens cDNA clone IMAGE:1285407 3' similar to contains Alu repetitive element;. mRNA sequence. |
| 233300_at | 0.71 | CDNA FLJ11548 fis. clone HEMBA1002944 |
| 236196_at | 0.709 | CDNA FLJ44883 fis. clone BRAMY2036918 |
| 212060_at | 0.708 | U2-associated SR140 protein |
| 200779_at | 0.707 | activating transcription factor 4 (tax-responsive enhancer element B67) |
| 200089_s_at | 0.707 | ribosomal protein L4 |
| 202931_x_at | 0.706 | bridging integrator 1 |
| 241724_x_at | 0.705 | Transcribed sequence with moderate similarity to protein sp:P39195 (H.sapiens) ALU8_HUMAN Alu subfamily SX sequence contamination warning entry |
| 225020_at | 0.704 | DAB2 interacting protein |
| 205420_at | 0.704 | peroxisomal biogenesis factor 7 |
| 228379_at | 0.701 | nuclear transport factor 2 |
| 209416_s_at | 0.7 | Fzr1 protein |
| 205546_s_at | 0.699 | tyrosine kinase 2 |
| 215243_s_at | 0.698 | gap junction protein; transmembrane protein; Homo sapiens connexin 31 (GJB3) gene. complete cds. |
| 234032_at | 0.698 | predicted protein of HQ1550; Homo sapiens PRO1550 mRNA. partial cds. |
| 201423_s_at | 0.697 | cullin 4A |
| 227111_at | 0.697 | CDNA FLJ31099 fis. clone IMR321000230 |
| 203782_s_at | 0.697 | polymerase (RNA) mitochondrial (DNA directed) |
| 241364_at | 0.696 | od08b10.s1 NCI_CGAP_GCB1 Homo sapiens cDNA clone IMAGE:1367323 3'. mRNA sequence. |
| 226316_at | 0.696 | chromosome 13 open reading frame 10 |
| 218396_at | 0.696 | vacuolar protein sorting 13C (yeast) |
| 234998_at | 0.696 | CDNA clone IMAGE:5313062. partial cds |
| 202424_at | 0.696 | mitogen-activated protein kinase kinase 2 |
| 217941_s_at | 0.695 | erbb2 interacting protein |
| 230270_at | 0.694 | yw88f05.s1 Soares_placenta_8to9weeks_2NbHP8to9W Homo sapiens cDNA clone IMAGE:259329 3' similar to contains Alu repetitive element;. mRNA sequence. |
| 205004_at | 0.694 | NF-kappa B-repressing factor |
| 202828_s_at | 0.694 | matrix metalloproteinase 14 (membrane-inserted) |
| 205370_x_at | 0.694 | dihydrolipoamide branched chain transacylase (E2 component of branched chain keto acid dehydrogenase complex; maple syrup urine disease) |
| 225893_at | 0.693 | MRNA; cDNA DKFZp686D04119 (from clone DKFZp686D04119) |
| 227005_at | 0.693 | ribonuclease P (14kD) |
| 236462_at | 0.692 | hypothetical protein FLJ10201 |
| 223265_at | 0.691 | KIAA1720 protein |
| 212856_at | 0.691 | KIAA0767 protein |
| 228686_at | 0.691 | MRNA; cDNA DKFZp686D0374 (from clone DKFZp686D0374) |
| 1552426_a_at | 0.689 | BBP-like protein 2 |
| 200864_s_at | 0.689 | RAB11A. member RAS oncogene family |
| 225752_at | 0.687 | spastic paraplegia 6 (autosomal dominant) |
| 238722_x_at | 0.687 | ap34a09.x1 Schiller astrocytoma Homo sapiens cDNA clone IMAGE:1957240 3' similar to contains Alu repetitive element;contains MER12.b2 MER12 repetitive element ;. mRNA sequence. |
| 212208_at | 0.686 | thyroid hormone receptor associated protein 2 |
| 203883_s_at | 0.686 | KIAA0941 protein |
| 242712_x_at | 0.684 | Transcribed sequence with strong similarity to protein prf:2115329A (H.sapiens) 2115329A nucleoprotein Nup358 [Homo sapiens] |
| 229123_at | 0.681 | zinc finger protein 224 |
| 216251_s_at | 0.681 | KIAA0153 protein |
| 237333_at | 0.681 | intermediate filament protein syncoilin |
| 209609_s_at | 0.68 | mitochondrial ribosomal protein L9 |
| 1564637_a_at | 0.679 | hypothetical protein FLJ38426 |
| 226143_at | 0.679 | retinoic acid induced 1 |
| 225257_at | 0.677 | hypothetical protein MGC20255 |
| 1558996_at | 0.677 | forkhead box P1 |
| 221791_s_at | 0.677 | hypothetical protein HSPC016 |
| 228171_s_at | 0.674 | DKFZP434I216 protein |
| 229035_s_at | 0.673 | junctophilin 3 |
| 47571_at | 0.67 | zinc finger protein 236 |
| 201031_s_at | 0.669 | heterogeneous nuclear ribonucleoprotein H1 (H) |
| 242578_x_at | 0.666 | solute carrier family 22 (extraneuronal monoamine transporter). member 3 |
| 1552257_a_at | 0.663 | KIAA0153 protein |
| 232597_x_at | 0.659 | splicing factor. arginine/serine-rich 2. interacting protein |
| 217804_s_at | 0.659 | interleukin enhancer binding factor 3. 90kDa |
| 211300_s_at | 0.658 | tumor protein p53 (Li-Fraumeni syndrome) |
| 218555_at | 0.657 | anaphase promoting complex subunit 2 |
| 226428_at | 0.655 | transportin 2 (importin 3. karyopherin beta 2b) |
| 235434_at | 0.653 | CDNA FLJ42524 fis. clone BRACE3001384 |
| 213221_s_at | 0.653 | salt-inducible serine/threonine kinase 2 |
| 237895_at | 0.653 | Transcribed sequences |
| 201221_s_at | 0.651 | small nuclear ribonucleoprotein 70kDa polypeptide (RNP antigen) |
| 221860_at | 0.649 | heterogeneous nuclear ribonucleoprotein L |
| 204372_s_at | 0.649 | KH-type splicing regulatory protein (FUSE binding protein 2) |
| 223247_at | 0.645 | hypothetical protein MGC5309 |
| 233595_at | 0.641 | CDNA FLJ14279 fis. clone PLACE1005574 |
| 222761_at | 0.64 | basic. immunoglobulin-like variable motif containing |
| 203959_s_at | 0.64 | KIAA0478 gene product |
| 1554429_a_at | 0.639 | dystrophia myotonica-containing WD repeat motif |
| 212601_at | 0.634 | zinc finger. ZZ-type with EF hand domain 1 |
| 215600_x_at | 0.633 | Similar to hypothetical protein (LOC391537). mRNA |
| 238935_at | 0.632 | EST370545 MAGE resequences. MAGE Homo sapiens cDNA. mRNA sequence. |
| 222465_at | 0.63 | chromosome 15 open reading frame 15 |
| 235107_at | 0.62 | Msx-interacting-zinc finger |
| 1570210_x_at | 0.618 | Homo sapiens KIAA0685. mRNA (cDNA clone IMAGE:4250008). with apparent retained intron. |
| 223619_x_at | 0.616 | peroxisomal trans-2-enoyl-CoA reductase |
| 1559038_at | 0.613 | neural precursor cell expressed. developmentally down-regulated 5 |
| 209119_x_at | 0.612 | AV703465 ADB Homo sapiens cDNA clone ADBCHG08 5'. mRNA sequence. |
| 220079_s_at | 0.608 | synonyms: FLJ11328. FLJ20103. FLJ23054. FLJ23277. MGC14879; FLJ23277 protein; ubiquitin specific protease 31; go_function: peptidase activity [goid 0008233] [evidence IEA]; go_function: ubiquitin thiolesterase activity [goid 0004221] [evidence IEA]; go_function: cysteine-type endopeptidase activity [goid 0004197] [evidence IEA]; go_process: ubiquitin-dependent protein catabolism [goid 0006511] [evidence IEA]; Homo sapiens ubiquitin specific protease 48 (USP48). mRNA. |
| 236356_at | 0.602 | NADH dehydrogenase (ubiquinone) Fe-S protein 1. 75kDa (NADH-coenzyme Q reductase) |
| 221270_s_at | 0.597 | queuine tRNA-ribosyltransferase 1 (tRNA-guanine transglycosylase) |
| 227369_at | 0.595 | PAI-1 mRNA-binding protein |
| 209667_at | 0.594 | 601454201F1 NIH_MGC_66 Homo sapiens cDNA clone IMAGE:3857928 5'. mRNA sequence. |
| 235663_at | 0.592 | Transcribed sequences |
| 226045_at | 0.592 | fibroblast growth factor receptor substrate 2 |
| 202096_s_at | 0.592 | benzodiazapine receptor (peripheral) |
| 1554670_at | 0.588 | golgi associated. gamma adaptin ear containing. ARF binding protein 1 |
| 244197_x_at | 0.588 | wm22e03.x1 NCI_CGAP_Ut4 Homo sapiens cDNA clone IMAGE:2436700 3' similar to contains Alu repetitive element;. mRNA sequence. |
| 217945_at | 0.587 | BTB (POZ) domain containing 1 |
| 222163_s_at | 0.579 | hypothetical protein MGC5347 |
| 217653_x_at | 0.567 | Transcribed sequence with weak similarity to protein ref:NP_060312.1 (H.sapiens) hypothetical protein FLJ20489 [Homo sapiens] |
| 207598_x_at | 0.556 | X-ray repair complementing defective repair in Chinese hamster cells 2 |
| 209703_x_at | 0.533 | DKFZP586A0522 protein |
| 230387_at | 0.531 | Transcribed sequences |
| 231199_at | 0.529 | Transcribed sequence with weak similarity to protein pir:A32422 (H.sapiens) A32422 dihydrolipoamide S- |
| 206752_s_at | 0.524 | DNA fragmentation factor. 40kDa. beta polypeptide (caspase-activated DNase) |
| 231297_at | 0.519 | DOT1-like. histone H3 methyltransferase (S. cerevisiae) |
| 210686_x_at | 0.514 | synonyms: D10S105E. HGT.1. ML7. GDC. hML7. GDA. MGC39851; Homo sapiens solute carrier family 25 (mitochondrial carrier; Graves disease autoantigen). member 16. mRNA (cDNA clone IMAGE:3139311). complete cds. |
| 206169_x_at | 0.511 | synonyms: FLJ13787. KIAA1031. DKFZp434K0920; Rotavirus 'X' associated non-structural protein; Homo sapiens ubiquitous tetratricopeptide containing protein RoXaN (RoXaN). mRNA. |
| 242696_at | 0.503 | Transcribed sequences |
| 228933_at | 0.497 | Nance-Horan syndrome (congenital cataracts and dental anomalies) |
| 229966_at | 0.475 | Ewing sarcoma breakpoint region 1 |
| 244341_at | 0.469 | ob54g10.s1 NCI_CGAP_GCB1 Homo sapiens cDNA clone IMAGE:1335234 3'. mRNA sequence. |

| **N0 vs N1 up** |  |  |
| --- | --- | --- |
| **Probe Set ID** | **Fold Change** | **Gene Title** |
| 217320_at | 2.94 | Homo sapiens partial IGVH3 DP29 gene for immunoglobulin heavy chain V region. case 1. cell Mo VII 116. |
| 218676_s_at | 2.093 | phosphatidylcholine transfer protein |
| 201791_s_at | 1.985 | 7-dehydrocholesterol reductase |
| 208029_s_at | 1.962 | lysosomal associated protein transmembrane 4 beta |
| 214039_s_at | 1.915 | lysosomal associated protein transmembrane 4 beta |
| 210078_s_at | 1.842 | potassium voltage-gated channel. shaker-related subfamily. beta member 1 |
| 202874_s_at | 1.805 | ATPase. H+ transporting. lysosomal 42kDa. V1 subunit C. isoform 1 |
| 223721_s_at | 1.748 | J domain containing protein 1 |
| 235619_at | 1.738 | ankyrin repeat and SOCS box-containing 4 |
| 214651_s_at | 1.725 | homeo box A9 |
| 204610_s_at | 1.715 | hepatitis delta antigen-interacting protein A |
| 223597_at | 1.708 | intelectin 1 (galactofuranose binding) |
| 219402_s_at | 1.699 | hypothetical protein MGC3067 |
| 230728_at | 1.694 | UI-H-BW1-anz-e-11-0-UI.s1 NCI_CGAP_Sub7 Homo sapiens cDNA clone IMAGE:3084020 3'. mRNA sequence. |
| 200678_x_at | 1.692 | granulin |
| 203882_at | 1.676 | interferon-stimulated transcription factor 3. gamma 48kDa |
| 210471_s_at | 1.676 | potassium voltage-gated channel. shaker-related subfamily. beta member 1 |
| 201954_at | 1.648 | actin related protein 2/3 complex. subunit 1B. 41kDa |
| 202061_s_at | 1.644 | sel-1 suppressor of lin-12-like (C. elegans) |
| 229566_at | 1.637 | Similar to RIKEN cDNA 1100001G20 (LOC400610). mRNA |
| 204615_x_at | 1.629 | isopentenyl-diphosphate delta isomerase |
| 217738_at | 1.625 | pre-B-cell colony enhancing factor 1 |
| 210302_s_at | 1.623 | mab-21-like 2 (C. elegans) |
| 216041_x_at | 1.603 | granulin |
| 212956_at | 1.582 | qp61g12.x1 NCI_CGAP_Co8 Homo sapiens cDNA clone IMAGE:1927558 3'. mRNA sequence. |
| 201963_at | 1.579 | synonyms: ACS1. LACS. FACL1. FACL2. LACS1. LACS2; fatty-acid-Coenzyme A ligase. long-chain 2; long-chain acyl-CoA synthetase 2; fatty-acid-Coenzyme A ligase. long-chain 1; lignoceroyl-CoA synthase; palmitoyl-CoA ligase 2; long-chain acyl-CoA synthetase 1; paltimoyl-CoA ligase 1; go_function: long-chain-fatty-acid-CoA ligase activity [goid 0004467] [evidence TAS] [pmid 1531127]; go_function: magnesium ion binding [goid 0000287] [evidence IEA]; go_function: ligase activity [goid 0016874] [evidence IEA]; go_process: fatty acid metabolism [goid 0006631] [evidence NR]; go_process: digestion [goid 0007586] [evidence TAS] [pmid 1531127]; go_process: metabolism [goid 0008152] [evidence IEA]; Homo sapiens acyl-CoA synthetase long-chain family member 1 (ACSL1). mRNA. |
| 203519_s_at | 1.573 | UPF2 regulator of nonsense transcripts homolog (yeast) |
| 237721_s_at | 1.564 | ankyrin repeat and SOCS box-containing 4 |
| 218231_at | 1.555 | N-acetylglucosamine kinase |
| 201186_at | 1.552 | low density lipoprotein receptor-related protein associated protein 1 |
| 1552472_a_at | 1.551 | centaurin. beta 2 |
| 217796_s_at | 1.545 | hypothetical protein FLJ20657 |
| 223310_x_at | 1.525 | intracellular membrane-associated calcium-independent phospholipase A2 gamma |
| 224683_at | 1.524 | F-box only protein. helicase. 18 |
| 207485_x_at | 1.516 | butyrophilin. subfamily 3. member A1 |
| 214196_s_at | 1.511 | ceroid-lipofuscinosis. neuronal 2. late infantile (Jansky-Bielschowsky disease) |
| 217879_at | 1.51 | cell division cycle 27 |
| 227416_s_at | 1.509 | MADP-1 protein |
| 235579_at | 1.509 | splicing factor. arginine/serine-rich 2. interacting protein |
| 202582_s_at | 1.508 | RAN binding protein 9 |
| 205480_s_at | 1.499 | UDP-glucose pyrophosphorylase 2 |
| 238539_at | 1.498 | Hermansky-Pudlak syndrome 3 |
| 200096_s_at | 1.491 | ATPase. H+ transporting. lysosomal 9kDa. V0 subunit e |
| 223164_at | 1.483 | cerebral cavernous malformation 2 |
| 211404_s_at | 1.481 | amyloid beta (A4) precursor-like protein 2 |
| 202816_s_at | 1.478 | synovial sarcoma translocation. chromosome 18 |
| 210619_s_at | 1.472 | hyaluronoglucosaminidase 1 |
| 201143_s_at | 1.465 | eukaryotic translation initiation factor 2. subunit 1 alpha. 35kDa |
| 242571_at | 1.456 | CDNA FLJ40165 fis. clone TESTI2015962 |
| 223061_at | 1.455 | hypothetical protein MGC3234 |
| 235645_at | 1.449 | establishment factor-like protein |
| 220186_s_at | 1.448 | protocadherin LKC |
| 204550_x_at | 1.442 | glutathione S-transferase M1 |
| 201995_at | 1.436 | exostoses (multiple) 1 |
| 205645_at | 1.427 | RALBP1 associated Eps domain containing 2 |
| 219862_s_at | 1.425 | nuclear prelamin A recognition factor |
| 205084_at | 1.424 | B-cell receptor-associated protein 29 |
| 1554451_s_at | 1.417 | DnaJ protein |
| 213724_s_at | 1.416 | pyruvate dehydrogenase kinase. isoenzyme 2 |
| 231034_s_at | 1.41 | chromosome 6 open reading frame 63 |
| 223785_at | 1.408 | hypothetical protein FLJ10719 |
| 226684_at | 1.406 | chromosome 14 open reading frame 103 |
| 208729_x_at | 1.4 | major histocompatibility complex. class I. B |
| 219656_at | 1.397 | protocadherin 12 |
| 213695_at | 1.394 | paraoxonase 3 |
| 218642_s_at | 1.39 | coiled-coil-helix-coiled-coil-helix domain containing 7 |
| 225743_at | 1.383 | hypothetical protein MGC29784 |
| 202377_at | 1.376 | leptin receptor |
| 229025_s_at | 1.375 | hypothetical protein FLJ25059 |
| 218108_at | 1.375 | chromosome 14 open reading frame 130 |
| 201518_at | 1.371 | chromobox homolog 1 (HP1 beta homolog Drosophila ) |
| 226551_at | 1.37 | receptor (TNFRSF)-interacting serine-threonine kinase 1 |
| 206667_s_at | 1.37 | secretory carrier membrane protein 1 |
| 216620_s_at | 1.367 | Rho guanine nucleotide exchange factor (GEF) 10 |
| 219072_at | 1.362 | B-cell CLL/lymphoma 7C |
| 204820_s_at | 1.355 | butyrophilin. subfamily 3. member A3 |
| 220051_at | 1.354 | protease. serine. 21 (testisin) |
| 226453_at | 1.353 | AYP1 protein |
| 209329_x_at | 1.352 | Homo sapiens hypothetical protein MGC2198. mRNA (cDNA clone MGC:2198 IMAGE:3345790). complete cds. |
| 221821_s_at | 1.347 | hypothetical protein FLJ20436 |
| 239069_s_at | 1.345 | 602247087F1 NIH_MGC_62 Homo sapiens cDNA clone IMAGE:4332477 5'. mRNA sequence. |
| 201087_at | 1.341 | Paxillin (PXN). mRNA |
| 217975_at | 1.337 | pp21 homolog |
| 52940_at | 1.336 | single Ig IL-1R-related molecule |
| 213208_at | 1.336 | KIAA0240 |
| 208957_at | 1.335 | thioredoxin domain containing 4 (endoplasmic reticulum) |
| 215891_s_at | 1.334 | GM2 ganglioside activator protein |
| 210285_x_at | 1.333 | Wilms tumor 1 associated protein |
| 218034_at | 1.333 | tetratricopeptide repeat domain 11 |
| 203174_s_at | 1.332 | ADP-ribosylation factor related protein 1 |
| 209432_s_at | 1.332 | cAMP responsive element binding protein 3 |
| 202855_s_at | 1.33 | solute carrier family 16 (monocarboxylic acid transporters). member 3 |
| 203402_at | 1.33 | potassium voltage-gated channel. shaker-related subfamily. beta member 2 |
| 203117_s_at | 1.329 | ubiquitin specific protease 52 |
| 225885_at | 1.329 | MSTP105 (MST105) mRNA. complete cds |
| 228244_at | 1.327 | CDNA FLJ26676 fis. clone MPG03726 |
| 207821_s_at | 1.325 | PTK2 protein tyrosine kinase 2 |
| 223687_s_at | 1.324 | cDNA for differentially expressed CO16 gene |
| 227836_at | 1.324 | wm20c09.x1 NCI_CGAP_Ut4 Homo sapiens cDNA clone IMAGE:2436496 3'. mRNA sequence. |
| 1556180_at | 1.324 | Clone IMAGE:5272902. mRNA |
| 222657_s_at | 1.324 | hypothetical protein FLJ11011 |
| 220775_s_at | 1.323 | ubiquitin-conjugating enzyme E2-like |
| 204879_at | 1.321 | lung type-I cell membrane-associated glycoprotein |
| 203227_s_at | 1.32 | transmembrane 4 protein; go_component: membrane fraction [goid 0005624] [evidence TAS] [pmid 8134123]; go_component: integral to plasma membrane [goid 0005887] [evidence TAS] [pmid 8134123]; go_process: positive regulation of cell proliferation [goid 0008284] [evidence TAS] [pmid 8134123]; Homo sapiens sarcoma amplified sequence (SAS). mRNA. |
| 210431_at | 1.318 | alkaline phosphatase precursor (EC 3.1.31.); Human alkaline phosphatase (ALP-1) mRNA. complete cds. |
| 200049_at | 1.316 | MYST histone acetyltransferase 2 |
| 227124_at | 1.313 | CDNA FLJ26369 fis. clone HRT06001 |
| 204274_at | 1.311 | estrogen receptor binding site associated. antigen. 9 |
| 215690_x_at | 1.31 | GPAA1P anchor attachment protein 1 homolog (yeast) |
| 226478_at | 1.309 | CDNA FLJ34764 fis. clone NT2NE2002311 |
| 218109_s_at | 1.309 | hypothetical protein FLJ14153 |
| 218417_s_at | 1.304 | hypothetical protein FLJ20489 |
| 202829_s_at | 1.303 | synaptobrevin-like 1 |
| 224809_x_at | 1.302 | TERF1 (TRF1)-interacting nuclear factor 2 |
| 200918_s_at | 1.302 | signal recognition particle receptor ('docking protein') |
