# Supplementary material for: Vascular CXCR4 Expression – a Novel Antiangiogenic Target in Gastric Cancer?
Source: PLoS One. 2010 Apr 8;5(4):e10087. doi: 10.1371/journal.pone.0010087 (PMC2851611; doi:10.1371/journal.pone.0010087)
Supplement: Table S2 — Differentially expressed GPCRs and GPCR-related genes in the primary tumors of node-negative (N0) vs. node-positive (N1) intestinal type primary gastric carcinomas based on microarray analysis (fold change factor >1.5). (0.11 MB DOC) [file pone.0010087.s002.doc]

**Table S2:** Differentially expressed GPCRs and GPCR-related genes in the primary tumors of node-negative (N0) vs. node-positive (N1) intestinal type primary gastric carcinomas based on microarray analysis (fold change factor >1.5).

| **Probe Set ID** | **Gene Symbol** | **Gene Title** | **Node positive normalized** | **Node negative normalized** |
| --- | --- | --- | --- | --- |
| 203108_at | GPRC5A | G protein-coupled receptor, family C, group 5, member A | 0,605 (0,31 to 1,174) | 0,916 (0,591 to 1,348) |
| 210279_at | GPR18 | G protein-coupled receptor 18 | 1 | 1 |
| 223620_at | GPR34 | G protein-coupled receptor 34 | 1,723 (0,911 to 4,829) | 0,957 (0,717 to 1,708) |
| 1555989_at | GPR135 | G protein-coupled receptor 135 | 1,668 (0,959 to 8,085) | 1,461 (0,959 to 3,386) |
| 223423_at | GPR160 | G protein-coupled receptor 160 | 1,988 (0,464 to 12,7) | 0,937 (0,446 to 1,821) |
| 230369_at | GPR161 | G protein-coupled receptor 161 | 1,551 (0,904 to 3,796) | 1,315 (0,904 to 2,711) |
| 207651_at | GPR171 | G protein-coupled receptor 171 | 1,269 (0,957 to 2,961) | 1,519 (1,043 to 2,068) |
|  |  |  |  |  |
| 1555890_at | OR2A7 | Olfactory receptor, family 2, subfamily A, member 7 | 1,544 (1 to 2,559) | 1 |
| 1564333_a_at | SORCS2 | Sortilin-related VPS10 domain containing receptor 2 | 1 | 1,709 (1 to 4,994) |
| 202307_s_at | TAP1 | transporter 1, ATP-binding cassette, sub-family B (MDR/TAP) | 0,839 (0,405 to 1,278) | 0,6 (0,335 to 1,456) |
| 204036_at | EDG2 | endothelial differentiation, lysophosphatidic acid G-protein-coupled receptor, 2 | 1,512 (0,933 to 2,918) | 1,14 (0,728 to 2,424) |
| 205119_s_at | FPR1 | formyl peptide receptor 1 /// formyl peptide receptor 1 | 1,614 (0,854 to 3,817) | 1,009 (0,854 to 1,114) |
| 205226_at | PDGFRL | platelet-derived growth factor receptor-like | 1,792 (1 to 4,002) | 1 |
| 205357_s_at | AGTR1 | Angiotensin II receptor, type 1 | 1,495 (1 to 4,995) | 1,628 (1 to 4,311) |
| 206631_at | PTGER2 | prostaglandin E receptor 2 (subtype EP2), 53kDa | 1,582 (1 to 4,773) | 1,541 (1 to 3,656) |
| 208335_s_at | DARC | Duffy blood group, chemokine receptor | 2,151 (1 to 7,836) | 1,034 (1 to 1,104) |
| 213880_at | LGR5 | leucine-rich repeat-containing G protein-coupled receptor 5 | 1,602 (0,6 to 2,861) | 1,096 (0,6 to 3,653) |
| 214348_at | TACR2 | tachykinin receptor 2 | 1,667 (1 to 7,721) | 1,486 (1 to 3,282) |
| 214560_at | FPRL2 | formyl peptide receptor-like 2 | 1,514 (0,794 to 3,419) | 0,925 (0,794 to 1,255) |
| 220108_at | GNA14 | guanine nucleotide binding protein (G protein), alpha 14 | 1 | 2,01 (1 to 3,438) |
| 227819_at | LGR6 | leucine-rich repeat-containing G protein-coupled receptor 6 | 1,001 (0,994 to 1,022) | 1,592 (0,994 to 2,793) |
| 242871_at | PAQR5 | progestin and adipoQ receptor family member V | 1 | 1,921 (1 to 3,241) |
|  |  |  |  |  |
| 203915_at | CXCL9 | chemokine (C-X-C motif) ligand 9 | 0,574 (0,162 to 1,395) | 1,178 (0,384 to 4,122) |
| 203666_at | CXCL12 | chemokine (C-X-C motif) ligand 12 (stromal cell-derived factor 1) | 1,908 (0,693 to 5,31) | 1,188 (0,693 to 3,151) |
| 209687_at | CXCL12 | chemokine (C-X-C motif) ligand 12 (stromal cell-derived factor 1) | 2,125 (0,571 to 9,205) | 0,781 (0,335 to 2,705) |
| 216598_s_at | CCL2 | chemokine (C-C motif) ligand 2 | 1,705 (0,667 to 2,852) | 0,63 (0,337 to 1,333) |
|  |  |  |  |  |
| 1555229_a_at | C1S | complement component 1, s subcomponent | 1,516 (0,564 to 3,914) | 0,65 (0,36 to 0,963) |
| 209906_at | C3AR1 | complement component 3a receptor 1 | 1,54 (0,897 to 3,519) | 0,875 (0,465 to 1,405) |
|  |  |  |  |  |
| 1553613_s_at | FOXC1 | forkhead box C1 | 0,881 (0,766 to 1,032) | 1,519 (0,88 to 2,496) |
| 1553989_a_at | ATP6V1C2 | ATPase, H+ transporting, lysosomal 42kDa, V1 subunit C2 | 1,523 (1 to 3,542) | 1,657 (1 to 4,549) |
|  |  |  |  |  |
| 202388_at | RGS2 | regulator of G-protein signalling 2, 24kDa | 1,513 (0,654 to 3,32) | 1,293 (0,884 to 2,653) |
| 209325_s_at | RGS16 | regulator of G-protein signalling 16 | 1,55 (0,935 to 3,396) | 1,223 (0,935 to 1,837) |
|  |  |  |  |  |
| 202458_at | PRSS23 | protease, serine, 23 | 1,103 (0,614 to 1,764) | 0,571 (0,306 to 1,223) |
| 203914_x_at | HPGD | hydroxyprostaglandin dehydrogenase 15-(NAD) | 0,806 (0,374 to 1,132) | 1,619 (0,933 to 3,256) |
| 206023_at | NMU | Neuromedin U | 1,039 (0,39 to 4,601) | 0,626 (0,39 to 1,61) |
| 206339_at | CART | cocaine- and amphetamine-regulated transcript | 1,399 (1 to 3,832) | 1,508 (1 to 3,432) |
| 206577_at | VIP | vasoactive intestinal peptide | 1,53 (1 to 5,484) | 1,557 (1 to 3,772) |
| 208702_x_at | APLP2 | amyloid beta (A4) precursor-like protein 2 | 1,064 (0,842 to 1,19) | 0,653 (0,506 to 0,909) |
| 209758_s_at | MFAP5 | microfibrillar associated protein 5 | 1,227 (0,928 to 1,745) | 0,635 (0,463 to 1,072) |
| 210338_s_at | HSPA8 | heat shock 70kDa protein 8 | 1,056 (1,024 to 1,113) | 0,663 (0,553 to 0,848) |
| 211506_s_at | IL8 | interleukin 8 | 0,815 (0,222 to 1,917) | 0,614 (0,222 to 1,948) |
| 211548_s_at | HPGD | hydroxyprostaglandin dehydrogenase 15-(NAD) | 0,821 (0,353 to 1,289) | 1,519 (0,845 to 3,065) |
| 211695_x_at | MUC1 | mucin 1, cell surface associated /// mucin 1, cell surface associated | 0,993 (0,798 to 1,282) | 1,632 (0,885 to 2,276) |
| 213287_s_at | KRT10 | keratin 10 (epidermolytic hyperkeratosis; keratosis palmaris et plantaris) | 1,524 (0,839 to 6,622) | 0,945 (0,847 to 1,061) |
| 213710_s_at | CALM1 | Calmodulin 1 (phosphorylase kinase, delta) | 1,679 (0,999 to 2,432) | 0,948 (0,923 to 1,001) |
| 213714_at | CACNB2 | calcium channel, voltage-dependent, beta 2 subunit | 1,258 (1 to 2,505) | 1,582 (1 to 3,702) |
| 216510_x_at | IFI6 /// IGHA1 /// IGHD /// IGHG1 /// IGHM /// IL8 /// EXOC7 /// ZCWPW2 | interferon, alpha-inducible protein 6 /// immunoglobulin heavy constant alpha 1 /// immunoglobulin heavy constant delta /// immunoglobulin heavy constant gamma 1 (G1m marker) /// immunoglobulin heavy constant mu /// interleukin 8 /// exocyst complex component 7 /// zinc finger, CW type with PWWP domain 2 | 2,153 (0,706 to 8,186) | 1,21 (0,818 to 2,213) |
| 218963_s_at | KRT23 | keratin 23 (histone deacetylase inducible) | 4,182 (0,83 to 135,1) | 1,9 (0,83 to 9,963) |
| 223862_at | GHRL | ghrelin/obestatin preprohormone | 1 | 2,143 (1 to 9,839) |
| 224325_at | FZD8 | frizzled homolog 8 (Drosophila) /// frizzled homolog 8 (Drosophila) | 1,554 (1 to 2,836) | 1,144 (1 to 1,499) |
| 229274_at | GNAS | GNAS complex locus | 1,194 (1 to 2,03) | 1,919 (1 to 4,539) |
| 234792_x_at | IGHA1 /// IGHV2-70 | immunoglobulin heavy constant alpha 1 /// immunoglobulin heavy variable 2-70 | 1,6 (0,832 to 4,489) | 1,065 (0,782 to 1,468) |

**Table S3:** Patient characteristics of RT-PCR validation sample set.

| **Gastric carcinoma** | **n** |
| --- | --- |
|  |  |
|  |  |
| **total** | 37 |
|  |  |
| **Histology** |  |
| intestinal type | 37 |
| diffuse type | - |
|  |  |
| **Age, years** |  |
| ≤ 65 | 17 |
| > 65 | 20 |
|  |  |
| **Gender** |  |
| men | 18 |
| women | 19 |
|  |  |
| **T category** |  |
| pT1 | 3 |
| pT2a | 6 |
| pT2b | 17 |
| pT3 | 9 |
| pT4 | 2 |
|  |  |
| **Lymph nodes** |  |
| patients without metastases | 12 |
| patients with metastases | 25 |
|  |  |
| **Grade** |  |
| G1 | - |
| G2 | 21 |
| G3 | 14 |
| G4 | - |
| na | 2 |
|  |  |
