# Supplementary material for: Vascular CXCR4 Expression – a Novel Antiangiogenic Target in Gastric Cancer?
Source: PLoS One. 2010 Apr 8;5(4):e10087. doi: 10.1371/journal.pone.0010087 (PMC2851611; doi:10.1371/journal.pone.0010087)
Supplement: Table S3 — Patient characteristics of RT-PCR validation sample set. (0.06 MB DOC) [file pone.0010087.s003.doc]

**Table S3:** Patient characteristics of RT-PCR validation sample set.

| **Gastric carcinoma** | **n** |
| --- | --- |
|  |  |
|  |  |
| **total** | 37 |
|  |  |
| **Histology** |  |
| intestinal type | 37 |
| diffuse type | - |
|  |  |
| **Age, years** |  |
| ≤ 65 | 17 |
| > 65 | 20 |
|  |  |
| **Gender** |  |
| men | 18 |
| women | 19 |
|  |  |
| **T category** |  |
| pT1 | 3 |
| pT2a | 6 |
| pT2b | 17 |
| pT3 | 9 |
| pT4 | 2 |
|  |  |
| **Lymph nodes** |  |
| patients without metastases | 12 |
| patients with metastases | 25 |
|  |  |
| **Grade** |  |
| G1 | - |
| G2 | 21 |
| G3 | 14 |
| G4 | - |
| na | 2 |
|  |  |
